# Physical Activity, Sitting Time, and Quality of Life among Breast and Gynaecology Cancer Survivors

**DOI:** 10.31557/APJCP.2021.22.8.2399

**Published:** 2021-08

**Authors:** Nadzirah Hanis Zainordin, Norimah A Karim, Mohd Razif Shahril, Ruzita Abd Talib

**Affiliations:** 1 *Nutritional Sciences Programme, Faculty of Health Sciences, Universiti Kebangsaan Malaysia, Jalan Raja Muda Abdul Aziz, Kuala Lumpur, Malaysia. *; 2 *Center for Community Health Studies (ReaCH), Faculty of Health Sciences, Universiti Kebangsaan Malaysia, Jalan Raja Muda Abdul Aziz, Kuala Lumpur, Malaysia. *; 3 *Department of Nutrition and Dietetics, School of Health Sciences, International Medical University, Bukit Jalil, Kuala Lumpur, Malaysia. *; 4 *Center for Healthy Ageing and Wellness (H-CARE), Faculty of Health Sciences, Universiti Kebangsaan Malaysia, Jalan Raja Muda Abdul Aziz, Kuala Lumpur, Malaysia. *

**Keywords:** Breast Cancer, gynaecology cancer, survivor, physical activity, sitting, quality of life

## Abstract

**Background:**

Increasing physical activity and reducing sitting time was recommended to cancer survivors after cancer treatment for sustained health and to enhance the quality of life. This study aimed to determine the association of physical activity and sitting time with quality of life among the Malay breast and gynaecological cancer survivors.

**Methods:**

A cross-sectional study was conducted among 95 breast and gynaecology cancer survivor subjects. The Malay International Physical Activity Questionnaire (IPAQ) was used to assess physical activity and sitting time. Quality of life was assessed using the Malay EORTC QLQ-C30 questionnaire. Sociodemographic, clinical characteristics and anthropometric measurements were also obtained in this study.

**Results:**

The mean age of the subject was 51.8 ± 7.7 years old and the duration of survivorship was 4.3 ± 3.4 years. A total of 76.8% of subjects were categorized as having low physical activity level with a mean MET 403.5 ± 332.7 minutes/week and sitting time of 416.9 ± 151.0 minutes/day. Overall, subjects aged 50 years and above (p=0.006), widowed (p=0.032), retired (p=0.029) and had other non-communicable diseases (p=0.005) showed lower levels of physical activity. Increased physical activity had a positive effect on physical function (r=0.2, p=0.038), reduced insomnia (r=-0.3, p<0.001) and constipation symptoms (r=-0.3, p=0.012) domains of quality of life. The longer the sitting period showed more severe insomnia symptoms (r=0.2, p=0.03) but improved social function (r=0.2, p=0.012).

**Conclusions:**

Increasing physical activity and reducing sitting time have a positive effect on the quality of life of cancer survivors. The focus of health education should be prioritized to older adults (50 years and above), widows, retirees, and those with other comorbidities as they are at risk of being not physically active.

## Introduction

Early detection of cancer and adherence to treatment regimen has significantly improved the survival rate of cancer patients (WCRF/AICR, 2018a). In the United States of America, it has been estimated that the number of cancer survivor will increase from 16.9 million in 2019 to 22.1 million in 2030 (Miller et al., 2019). In Malaysia, the first population-based cancer survivor cohort project named Malaysia Study on Cancer Survival (MyScan) from 2007 to 2011 had recorded 72,884 cases and 35.7% of these survivors had lived exceeding the 5-year survivorship (National Cancer Registry [NCR], 2018). Among female cancer, especially breast, cervical, ovarian and uterine corpus, the survival rate in 5 years had been reported to be at 50% (NCR, 2018). 

Cancer survivors are struggling with treatment-related side effects that are affecting their quality of life after undergoing cancer treatment (Amarsheda and Bhise 2021). Long-term side effects usually reported include fatigue, pain, cardiotoxicity, neuropathy, cognitive dysfunction, and sexual and mental health issues (Gegechkori et al., 2017). Measurement of the quality of life is important in determining patients’ perceptions, the impact of disease, side effects of treatment, treatment effectiveness, and assessment of the patient’s psychosocial well-being (Kypriotakis et al., 2016). Furthermore, this assessment is useful in providing additional information to health professionals for them to plan further treatment (Quinten et al., 2014). Positive thinking, appropriate social relationships, economic support, cancer and self-care education, mental and physical well-being, religious beliefs, and being physically active will help cancer survivors improve their quality of life (Zaker et al., 2019).

Cancer survivors tend to be inactive and live a sedentary lifestyle after receiving the treatment (Sweegers et al., 2019). Fassier et al., (2016) showed that the sitting time had increased among cancer survivors especially in women, older patients, and those sedentary employees. Similarly, Trinh et al., (2015) and Kim et al., (2013) also reported a significantly longer duration of sedentary behaviour among cancer survivors compared to non-cancer participants. The side effects of cancer treatment like pain, fatigue, stress and insomnia might aggravate the survivors to remain sedentary throughout the day (Trinh et al., 2015). Besides, the beliefs that cancer survivors should rest longer, stay at home and avoid intense activity were factors leading to higher sedentary behaviour (Hong et al., 2019). Consequences from low physical activity and sedentary behaviour among cancer survivors may increase their risk of developing the non-communicable disease (NCD) or aggravate the condition of existing NCD (World Health Organization [WHO] (2010). Zaorsky et al., (2017) also concluded sedentary lifestyle increased the risk of premature death in cancer survivors. In addition, being at an inactive level and long sedentary periods lower the quality of life (Nurnazahiah et al., 2020), the decline in physiological system, initially cardio-respiratory, muscular systems, increase risk of joint immobility, osteoporosis, inbalance, paresthesia and lower back pain (Amarsheda and Bhise, 2021).

The American College of Sports Medicine (ACSM) roundtable discussion had concluded that cancer survivors shall remain physically active and resume routine activities to maintain overall health status and improve longevity (Patel et al., 2019). Studies also showed that physical activity has the potential to reduce the risk of mortality (Spei et al., 2019) and cancer recurrence (Cormie et al., 2017). Physical activity can reduce the risk of cancer by improving insulin sensitivity and reducing fasting insulin levels, decreasing oxidative stress and enhancing DNA repair mechanisms (WCRF/AICR 2018b). The American Cancer Society (Rock et al., 2020) has also recommended in the Guideline for Diet and Physical Activity for Cancer Prevention that cancer survivors shall reduce their sedentary behaviour. 

In Malaysia, most of the studies demonstrated that the level of physical activity among cancer survivors was low (Rufa’i., Yen and Muda 2019; Sulaiman et al., 2017; Yaw et al., 2014; Loh, Chew and Quek 2013; Zalina, Lee and Kandiah 2012) although physical activity is an important factor in improving the quality of life among cancer survivors (Nurnazahiah et al., 2020; Rufa’I et al., 2019; Loh, Lee, and Murray 2014). However, limited studies investigate the sitting time and its effect on the cancer survivors’ quality of life among breast and gynaecology cancer survivors. In addition, socio-cultural practices and obstacles which vary according to race (Loh et al., 2011) make this study focused on the Malays who form the majority of the population in Malaysia. Furthermore, among the ethnic groups in Malaysia, the survival rate was lowest among the Malays compared to the Chinese and Indian (NCR, 2018). Therefore, this study aimed to investigate the relationship between physical activity and sitting time on the quality of life among the Malay breast and gynaecological cancer survivors. 

## Materials and Methods

This cross-sectional study was conducted in the outpatient Radiotherapy and Oncology Clinic, and Gynaecology Clinic at the Hospital Kuala Lumpur (HKL) and Hospital Canselor Tuanku Muhriz Hospital (HCTM). These two hospitals are the referral medical centre for cancer patients in the central region of Malaysia. The subjects were recruited using convenience sampling based on the following criteria: Malaysian, Malays, aged between 18 to 65 years old, diagnosed with either breast or gynaecological cancer (cervical, ovarian, uterine, vaginal, or vulvar) at stage I, II or III. The subjects had completed all clinical treatment for more than six months and without a history of cancer recurrence. The subjects were selected for this study was part of a development of education modules for Malay cancer survivor related with food intake, activity physical and psychosocial.

All prospective subjects’ medical records were reviewed and shortlisted based on the inclusion criteria. The shortlisted subjects were invited to join the study while they were waiting for their consultation at the clinic. Written informed consent was obtained before the study commenced. This study excluded those who were pregnant, had a physical disability, or is active athlete during the data collection period, as this could affect the study outcomes. 

*Ethical Approval*

This study has been approved by the Medical Research and Ethics Committee, Ministry of Health Malaysia (NMRR-15-1435-26831) and Universiti Kebangsaan Malaysia Ethics Committee (NN-049-2015).

*Instruments*

A set of interviewer-administered questionnaire was used to collect subjects’ data. Socio-demographic information including age, marital status, education level, employment status and monthly household income was obtained. Clinical characteristics e.g. type of cancer, staging, duration of diagnosis, treatment type, and comorbidities were obtained from the medical records. Subjects’ heights were measured using the portable stadiometer (SECA Model 213, Germany) to the nearest 0.1 cm. The weight was measured with a digital weighing scale (SECA Model 880, Germany) to the nearest 0.1 kg. Body mass index (BMI) was deduced from the weight (kg) per square of height (m^2^) and categorized according to the WHO (1995) classification.

*Physical activity assessment*

The intensity of physical activity and sitting time was assessed by the International Physical Activity Questionnaire (IPAQ) (Craig et al., 2003). The short version of IPAQ (Short-IPAQ) was used to gauge the time spent being physically active in the last seven days. It is seven items measuring four domains of physical activity intensity: vigorous (referred to activities requiring hard physical effort lead to much harder than normal breathing); moderate (referred to moderate physical activities requiring harder than normal breathing); walking and sitting. For each activity domain, the subjects were required to report on the frequency during the last seven days and duration (minutes/hours) usually spent on one of those days in doing activities related to each domain. 

Based on the IPAQ scoring protocol, metabolic equivalent task minutes per week (MET-minutes/week) were used to calculate the physical activity level and intensity. To compute the MET scores for each activity, the raw score of total minutes spent over the last 7 days on vigorous activity, moderate-intensity activity and walking were multiplied by 8.0, 4.0, and 3.3, respectively. The total physical activity score was generated by summing up all the three sub-components of MET. MET was categorised into three physical activity levels; low (MET <600 min/week), moderate (MET 600-1,500 min/week) and high (MET>1500 min/week). The total sitting time (hours per day) was calculated based on subjects’ recall of the total time they spent sitting or lying down excluding time spent sleeping on a typical day. Total daily sitting time was used as a reference for the overall sedentary behaviour. The Malay version of IPAQ has been validated previously by Chu and Moy (2012).

*Quality of Life*

The EORTC QLQ-C30 questionnaire developed by the European Organization for Research and Treatment of Cancer (Fayers et al., 2001) was used to evaluate the subjects’ quality of life. The EORTC QLQ-C30 is a multi-dimensional cancer-specific questionnaire developed to evaluate the QoL of cancer patients. The assessment is based on past one-week experiences. The questionnaire has three scales: functional scale (physical, role, emotional, cognitive, and social), symptoms scale (nausea/vomiting, pain, dyspnoea, insomnia, appetite loss, constipation, diarrhoea, and financial difficulties) and global health scale (GHS). The items in both the functional and symptom scale are rated on a four Likert scale with 1 being “not at all” to 4 beings “very much”. On the other hand, the GHS is rated with a seven Likert scale with 1 being “very poor” to 7 being “excellent”. According to the scoring manual guideline of EORTC QLQ-C30 (Fayers et al., 2001), the raw score of each subscale is linearly transformed to standardize score ranging from 0 to 100. A higher score in the functional and GHS scale indicates better QoL. However, a higher score on the symptom scale reveals greater severity in symptom. This study employed the Malay version of EORTC QLQ-C30 which has been previously validated by Yusoff et al., (2010). 

*Statistical Analysis*

The IBM SPSS Statistics for Windows, Version 22 (IBM Corp, Armonk, NY, USA) software was used to analyse the data. Descriptive statistic was used to describe the socio-demographic, clinical characteristic, anthropometric, physical activity and QoL data. Parametric test such as independent t-test and one-way ANOVA were used to compare mean differences between breast and gynaecological cancer subjects on the age, duration of survivorship, BMI, MET and sitting time. The Chi-Square test was used to compare categorical differences between breast and gynaecological cancer subjects on the group of socio-demographic, clinical characterises and BMI categories. Spearman’s Rho correlation was conducted to explore the relationship between physical activity (MET) and sitting time towards EORTC QLO-C30. EORTC QLQ-30 were reported as non-normal distribution. The level of significance was set at p<0.05.

## Results

A total of 95 subjects, aged ranged between 34-65 with a mean of 51.8 ± 7.7 years old participated in this study (Table 1). The majority of them were diagnosed with breast cancer (65.6%), married (73.7%), had a secondary or lower educational status (54.7%), working (50.5%) and had a monthly household income between MYR 3000-4999 (30.5%). The mean duration since diagnosis with cancer were 4.3 ± 3.4 years. Clinical characteristic of a significant difference was observed in the cancer staging (p<0.001), type of treatment (p<0.001) and co-morbidity of NCD (p=0.043) between breast and gynaecology subjects. The mean BMI among all the subjects was 28.1±5.4 kg/m^2^ with almost equal distribution among the three categories of normal weight (34.7%), overweight (31.6%) and obese (33.7%). 

**Figure 1:**
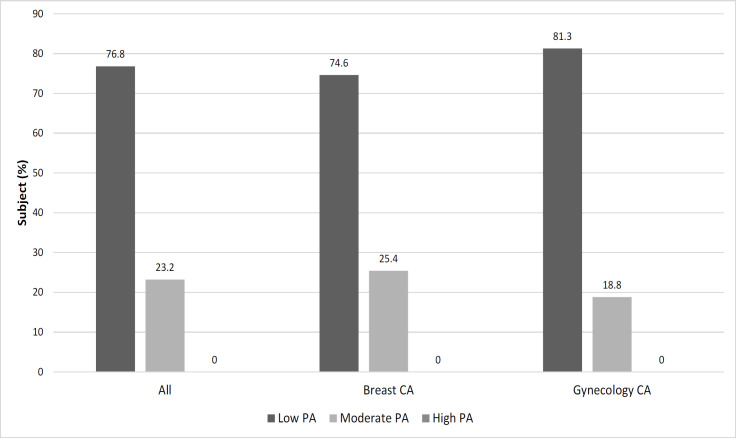
Level of Physical Activity According to the Type of Cancer

Table 2 illustrates the mean total MET was 403.5 ± 332.7 minutes/week and the mean total sitting time was 416.9 ± 151.0 minutes/day for all the subjects. Subjects who were aged 50 or above (p=0.006), widow (p=0.032), retired (p=0.029) and with at least one co-morbidity of NCD (p=0.005) showed a significantly lower level of physical activity compared to other groups. There were no significant differences in the sitting time among all the subjects based on the socio-demographic, clinical characteristic, and anthropometry measurement. Figure 1 shows a total of 76.8% of the subjects reported a low level of physical activity and none of them performed any high level of physical activity. 

The high mean score of GHS 79.5 ± 14.6 indicated the overall quality of life of the subjects was good (Table 3). A significant correlation was observed between MET and physical functioning scale (r=0.2, p=0.038). Subjects with higher physical activity showed better physical functioning. Significant negative correlations were found between MET with insomnia (r=-0.3, p<0.001) and constipation (r=-0.3, p=0.012) in the symptom scale. Lower physical activity would render the subjects experiencing higher adverse effects of insomnia and constipation. In terms of the correlation between sitting time and quality of life, subjects showed significant positive correlations with social functioning (r=0.3, p=0.012) and insomnia (r=0.2, p=0.03). Subjects with higher sitting time were having a better social function but experiencing higher insomnia symptom.

**Table 1:** Sociodemographic, Clinical Characteristic and BMI of the Subject (n=95)

Sociodemographic and Clinical Characteristics	All (n=95)	Breast Ca (n= 63)	Gynecology Ca (n= 32)	p value
Age (years) ^b^				
Min ± SD ^a^	51.8 ± 7.7	51.2 ± 6.9	53.9 ± 9.2	0.371
< 50	43 (45.3)	30 (47.6)	12 (40.6)	0.335
≥ 50	52 (44.7)	33 (53.4)	19 (59.4)	
Marital status ^b^				0.76
Single	13 (13.7)	8 (12.7)	5 (15.6)	
Married	70 (73.7)	46 (73.0)	24 (75.0)	
Widowed	12 (12.6)	9 (14.3)	3 (9.4)	
Education ^b^				0.19
Secondary and below	52 (54.7)	31 (49.2)	21 (65.6)	
Tertiary and below	43 (45.3)	32 (50.8)	11 (34.4)	
Employment status ^b^				0.122
Employed	48 (50.5)	36 (57.1)	14 (43.8)	
Unemployed	36 (37.9)	22 (57.1)	12 (37.5)	
Retireed	11 (11.6)	5 (7.9)	6 (18.8)	
Monthly household income (MYR) ^b^				0.55
< MYR 1,500	21 (22.1)	12 (19.0)	9 (28.1)	
MYR 1500.00 - 2999.00	17 (17.9)	12 (19.0)	5 (15.6)	
MYR 3000.00 - 4999.00	29 (30.5)	18 (28.6)	11 (34.4)	
≥ MYR 5000.00	28 (29.5)	21 (33.3)	7 (21.9)	
Duration of survivorship (years)b				
Mean ± SD ^a^	4.3 ± 3.4	3.9 ± 2.8	5.0 ± 4.3	0.187
< 1	12 (12.6)	6 (9.5)	6 (18.8)	0.417
1-2	29 (30.5)	21 (33.3)	8 (25.0)	
3-5	26 (27.4)	19 (30.2)	7 (21.9)	
> 5	28 (29.5)	17 (27.0)	11 (34.4)	
Stage of cancer ^b^				<0.001**
Stage I	33 (34.7)	11 (17.5)	22 (68.8)	
Stage II	41 (43.2)	34 (54.0)	7 (21.9)	
Stage III	21 (22.1)	18 (28.6)	3 (9.4)	
Types of gyne-cancer ^b^				
Ovarian	16 (50.0)	-	16 (50.0)	
Uterine	12 (37.5)	-	12 (37.5)	
Cervical/virginal/vulvar	4 (12.5)	-	4 (12.5)	
Type of treatment ^b^				<0.001**
Surgery	10 (10.5)	3 (4.8)	7 (21.9)	
Surgery + radiotherapy	11 (11.6)	4 (6.3)	7 (21.9)	
Surgery + chemotherapy	22 (23.2)	7 (11.1)	15 (46.9)	
Radiotherapy + Chemotherapy	2 (2.1)	0 (0)	2 (6.3)	
Surgery + Radiotherapy + Chemotherapy	50 (52.6)	49 (77.8)	1 (3.1)	
NCD^ b^				
No	59 (62.1)	44 (69.8)	15 (46.9)	0.043*
Yes	36 (37.9)	19 (30.1)	17 (53.1)	
BMI (kg/m^2^)^ b^				
Mean ± SD ^a^	28.1 ± 5.4	27.7 ± 5.3	28.5 ± 5.8	0.499
Normal	33 (34.7)	23 (36.5)	10 (31.3)	0.827
Overweight	30 (31.6)	20 (31.7)	10 (31.3)	
Obese	32 (33.7)	20 (31.7)	12 (37.5)	

Significant at *p<0.05; **p<0.001 using ^a^ Independent t-test and ^b^ Chi-square test

**Table 2:** Mean MET and Sitting Time According to Sociodemography, Clinical Characteristics and BMI

Characteristics	MET (minute/week)	p value	Sitting time (minute/day)	p value
Mean ± SD	403.5 ± 332.7		416.9 ± 151.0	
			[Median: 480.00]	
Age (years) ^a^				
< 50	504.3 ± 347.7	0.006*	411.6 ± 147.5	0.756
≥ 50	319.8 ± 297.9		421.3 ± 154.7	
Marital status ^b^				
Single	432.6 ± 240.5	0.032*	443.1 ± 170.6	0.056
Married	438.0 ± 350.2^ǂ^		397.0 ± 147.6	
Widowed	169.5 ± 213.4^ǂ^		505.0 ± 118.5	
Education ^a^				
Secondary and below	392.2 ± 350.7	0.722	425.9 ± 161.6	0.525
Tertiary and below	416.8 ± 313.1		406.0 ± 137.7	
Employment status ^b^				
Employed	450.3 ± 345.7^ǂ^	0.029*	413.7 ± 146.6	0.288
Housewife	415.5 ± 322.5		401.1 ± 157.8	
Retired	158.7 ± 186.5^ǂ^		482.7 ± 141.0	
Monthly household income (RM) ^b^				
< RM 1500	320.7 ± 292.6	0.078	471.4 ± 135.8	0.197
RM1500.00-RM 2999.00	297.1 ± 297.1		427.6 ± 174.3	
RM3000.00- RM4999.00	363.8 ± 363.8		40.7.6 ± 143.5	
> RM5000.00	323.7 ± 323.7		379.3 ± 148.8	
Type of cancer ^a^				
Breast	446.1 ± 334.3	0.079	416.8 ± 151.3	0.991
Gynecology	319.1 ± 317.9		417.1 ± 152.1	
Stage of cancer ^b^				
Stage I	380.1 ± 367.5	0.832	427.3 ± 155.2	0.885
Stage II	426.5 ± 342.4		410.0 ± 153.6	
Stage III	394.7 ± 260.3		414.3 ± 144.4	
Types of gyne-cancer ^b^				
Ovarian	383.4 ± 490.3	0.131	403.1 ± 186.5	0.846
Uterine	178.7 ± 195.1		425.0 ± 166.7	
Cervical/virginal/vulvar	483.0 ± 490.3		450.0 ± 141.1	
Duration of survivorship (years) ^b^				
< 1	395.9 ± 224.2	0.664	420.0 ± 125.3	0.843
1-2	484.6 ± 420.8		438.6 ± 140.8	
3-5	315.6 ± 265.1		403.0 ± 173.7	
> 5	403.4 ± 332.7		406.1 ± 153.1	
Type of treatment ^a^				
Chemotherapy	428.6 ± 342.1	0.543	411.6 ± 148.5	0.166
Without chemotherapy	314.4 ± 287.1		435.7 ± 160.9	
NCD ^a^				
No	472.4 ± 358.8	0.005*	424.1 ± 145.7	0.558
Yes	290.1 ± 250.4		405.3 ± 160.1	
BMI (kg/m^2^) ^b^				
Normal	386.2 ± 293.8	0.975	453.6 ± 142.3	0.148
Overweight	428.6 ± 373.9		379.3 ± 153.1	
Obese	397.3 ± 338.7		414.4 ± 153.6	

Significant at *p<0.05 using ^a^ Independent t-test and ^b^ ANOVA; ^ǂ ^post hoc.

**Table 3:** Correlation of MET Values and Sitting Time with QoL

Item EORTC	Score	MET (minute/week)		Sitting time (minute/day)	
		r_s_	p value	r_s_	p value
GHSa	79.5 ± 14.6	0.041	0.696	0.174	0.091
Functioning^a^					
Physical	83.8 ± 13.3	0.213	0.038*	-0.051	0.623
Role	72.9 ± 29.5	0.09	0.385	-0.01	0.921
Emotional	85.9 ± 17.8	0.094	0.367	-0.078	0.452
Cognitive	72.8 ± 19.6	0.129	0.212	-0.031	0.764
Social	96.5 ± 10.8	-0.146	0.157	0.257	0.012*
Symptoms^b^					
Fatigue	39.4 ± 21.8	-0.146	0.159	0.026	0.779
Nausea/ vomiting	27.7 ± 18.1	-0.155	0.135	0.017	0.874
Pain	27.7 ± 18.1	-0.155	0.135	0.017	0.874
Dyspnea	10.5 ± 21.3	-0.15	0.147	0.042	0.685
Insomnia	17.2 ± 26.1	-0.335	<0.001**	0.223	0.030*
Appetite loss	11.6 ± 23.2	-0.152	0.142	0.117	0.259
Constipation	12.3 ± 23.3	-0.256	0.012*	0.119	0.251
Diarrhea	8.8 ± 18.9	-0,055	0.598	-0.141	0.174
Financial difficulties	0.4 ± 3.4	-0.015	0.885	-0.067	0.52

Significant at *p<0.05, **p<0.001 using Spearmen-Rho test; ^a^, higher score represents better functioning or healthy level; ^b^, higher score represents a severe symptomatic problem

## Discussion

This study aimed to investigate the association between physical activity and sitting time on the quality of life of breast cancer and gynaecological cancer survivors. Overall, the finding showed that the majority of subjects had a low level of physical activity and a high level of daily sitting time. Among all the subjects, those who were older, widowed, retired, and had co-morbidities were more inclined to be physically inactive. In terms of the relationship between physical activity and quality of life, subjects with higher physical activity were better in the physical function domain and had a lower symptom of insomnia and constipation. On the other hand, the relationship between sitting time and quality of life revealed subjects with a longer sitting time displayed a better social function but experienced a higher symptom of insomnia.

The overall low level of physical activity among all the subjects in this study was consistent with other studies in the local setting (Rufa’i et al., 2019; Sulaiman et. al., 2017; Yaw et al., 2014; Loh et al., 2013; Zalina et al., 2012). This could be attributed to the physical pain experienced by most of the cancer survivors. The pain had been reported as a major barrier for cancer survivors’ participating in physical activity (Romero et al., 2018). As explained by Vlaeyen, and Linton (2000) in their dissemination of the fear-avoidance model of musculoskeletal pain, pain is an important factor in restricting an individual’s physical movement. Musculoskeletal pain experienced by an individual would install a perception of fear and worry within the individual which eventually leads to avoidance of physical activity (Romero et al., 2018).

In this finding, the demographic characteristics of the subjects which are older age, who are widower, retired and co-morbidities showed a lower level of physical activity were consistent with Su et al., (2019) study. The National Health and Morbidity Survey (NHMS) in Malaysia revealed widow had the lowest physical activity compared to single and married adults (National Institute of Health Malaysia 2019). Consistent with Jamil et al., (2015) who reported that the risk of widows practising a sedentary lifestyle was 1.1 times higher than a single individual. Widowhood can cause considerable disruption that brings distressing experiences, changes in responsibility and burden which could be prolonged and intense for some which may eventually become a barrier for physical activity (Dlugonski et al., 2017; Stahl and Schulz 2014). Furthermore, the social stigmatisation of widowhood has tended to place this group of people in social and financial inequity, causing physical activity to become an important activity (Li et al., 2016). Furthermore, NHMS reported less exposure to physical activity information through various mass media channels as one of the factors widows become inactive (National Institute of Health Malaysia 2019). 

The physical activity among cancer survivors with comorbidities was lower compared to those without co-morbidities in this study. This was similar to the NHMS findings showing that individuals with comorbidities e.g. diabetes, hypertension and dyslipidemia were more inclined to sedentary behaviour (Jamil et al., 2016). Besides, a study by Troeschel et al., (2018) found that additional comorbidities will increase the risk of inactivity by 26%. A qualitative study in exploring the barriers to physical activity among diabetic participants identified that physical functional limitation, pain, lack of time and accessibility to the facility were among the factors (Lidegaard et al., 2016). Public health or clinical interventions or individual counselling are needed among this target group because cancer survivors with NCD have a higher risk of mortality (Ranc et al., 2014).

Retired subjects of this study were at a low level of physical activity compared to those employed and housewives. The transition to the retirement phase expects an increase in physical activity after retirement because more time and focus is given to physical activity (McDonald et al., 2015). However, after retirement, it was found that a decrease of 10% level of physical activity and a continuous decrease of -4% to –6% occurred within 5 years (Jones et al., 2018). Retired groups showed low levels of physical activity due to old age, health problems, comorbid disease, and limited physical ability (Feng et al., 2016). Also, an increase in leisure-time physical activity after retirement causes the physical activity to slow down or delay and decreases physical activity in line with increasing age (Holstila et al., 2017).

Prolonged sedentary activity will increase the risk of mortality, type II diabetes, cardiovascular and recurrent cancer (Patel et al., 2019). The majority of subjects in this study were sedentary and had obesity problems; hence this will increase the risk of complications after cancer treatment. Compared to the NHMS study, the average sitting time of this study was higher with a median value of 480 minutes/day compared to 180 minutes/day (Jamil et al., 2016). This is supported by a National Health and Nutrition Examination Survey (NHANES) 2007–2010 study that showed cancer survivors allocated 8 hours and more to be sedentary compared to a healthy population (Kim et al., 2013). On average, cancer survivors increased their time spent during sitting and lying down by two to three hours daily after cancer treatment (Fassier et al., 2016) and by objective measurement cancer survivors spent about 10 hours of daily sedentary time (George et al., 2014; Nurnazahiah et al., 2020). Paxton et al., (2016) explained the main reasons for the high sedentary levels among cancer survivors are due to fatigue and pain factors. These problems encourage cancer survivors to choose to rest by watching television, doing daily activities by sitting and lying down (Paxton et al., 2016). However, a study found that prolonged sitting cannot reduce the fatigue experienced, on the contrary, they have even shown to have more severe fatigue symptom (Sweegers et al., 2019). Moreover, the stigma that cancer survivors need to rest, sit at home and avoid doing strenuous work is one of the reasons for the increased sedentary activity after cancer treatment (Hong et al., 2019).

In terms of quality of life, constipation is less reported by subjects with higher levels of physical activity. Constipation was recorded among 43% to 58% of cancer survivors and elevated incidence among palliative cancer patients (McMillan et al., 2013). Wickham (2017) indicated that three conditions may induce constipation were (i) organic (e.g: bacteria, neurologic disorder), (ii) functional (e.g: environment, stress, fatigue, low fibre and fluid intake) and (iii) drug-related effects (e.g: opioids, antibiotics). Chemotherapy-induced constipation (CIC) such as alkylating agents, antimetabolites, immunomodulating agents, mitotic inhibitors can cause cytotoxic side-effects that delay nerve ends in the gut and course bowel delayed (McQuade et al., 2016). Thomas, Holm and Al-Adhami (2014) explained the effect of doing regular physical activity indicates the ability to defecate and specified rectosigmoid patterns or a better amount of transit time in the colon. However, a meta-analysis study by Nakano et al., (2018) found that exercise intervention did not promote or suppress constipation among cancer survivors. This is supported by Tantawy et al., (2017) who showed that constipation problems may be influenced by the effects of tumours, opioids, as side effects of treatments with anticholinergic properties and not due to physical activity.

This study demonstrated that increase sitting time helps to improve the quality of life in social function. However, these findings were in contrast to a past study that showed that doing physical activities can enhance interactions and social relationships (Burke et al., 2017). Burke et al., (2017) explained an active lifestyle leads to feelings of being understood by others, having good social relationships and being more open to cancer. Cancer treatment had changed cancer survivors the physical, psychological and health status which influences their social functioning (Fong et al., 2016; Zaker et al. 2019). This situation encouraging social isolation among cancer survivors from the public (Hinzey et al., 2016) and spend more with sedentary activity. Although cancer survivors may have lost their usual social interaction, they have received support from their family and friends, which influences their emotional well-being (Amarsheda and Bhise 2021). In this study it is possible that the Malay subjects who have more sitting time, also allocate more time to spend with family and friends by eating, chatting, and resting activity. Additionally, the practice of less exposure to outside social relationships leads to a comfortable and safe condition for these cancer survivors. 

Increasing physical activity and reducing sitting time can lower insomnia symptoms are reported in this study. Insomnia can occur due to treatment side effects such as pain, fatigue, anxiety, stress, urinary incontinence, hot flushes and menopausal symptoms among cancer survivors (George et al., 2016; Tian et al., 2015). In general, cancer survivors experience a lower quality of sleep compared to the group that does not have cancer (Rafie et al., 2018). By doing physical activity as simple as walking can help improve the quality of sleep for cancer survivors (Chiu et al., 2015). Kline et al., (2017) explained by doing physical activities helps reduce distractions and difficulty breathing during sleep. The effects of insomnia and sedentary are interrelated because insomnia will increase the symptoms of fatigue, emotional disturbances and daily activity disorders as well as induce sedentary activity (George et al., 2016).

The limitations of this study are due to the natural disadvantages of a cross-sectional study and a small sample size. Physical activity information was obtained using self-report questionnaires that led to over-or under-reporting of physical activity intensity and most of the reporting will be influenced by memory. The use of objective measurement tools on physical activities such as accelerometer could provide more accurate physical activity information but will be costly. Besides, this study does not have information related to environmental factors that may affect the lifestyle of cancer survivors such as the location of residence, facilities for physical activity and support from people surroundings. Details of the daily activities performed by the subjects were also not identified in this study. Knowing the daily activities can help in identifying the obstacles experienced by the subject to perform physical activities. Modifications and matching of responsibilities as mothers, wives and employees can be planned to enable the subject to remain active despite being busy with daily affairs. 

In conclusion, breast cancer and gynaecological cancer survivors are at a low and sedentary level of physical activity after cancer treatment. The recommendations of ACSM and WCRF/AICR to remain active after cancer treatment were not achieved by the subjects of this study. The 50 years above, widows, retirees, and those with comorbidities group of the Malay cancer survivors at high risk for low levels of physical activity. Increased physical activity and reduced sitting periods have a positive effect on the quality of life in terms of physical function, symptoms of constipation and insomnia. Physical activity is one of the key factors that can change the health of cancer survivors, therefore, intervention to increase physical activity appropriate to the ability of cancer survivors should be implemented. 

## Author Contribution Statement

The authors confirm contribution to the paper as follows: Author N. H. Z. -Conception or design of the work, data collection, data analysis and interpretation, and drafting the article. Authors: N. A. K; M. R. S; R. A.T. - Conception or design of the work and critical revision of the article. All authors reviewed the results and approved the final version of the manuscript.

## Data Availability

This study is part of the approved student thesis and data is available upon request.
